# Evaluation of immune responses to *Brucella* vaccines in mouse models: A systematic review

**DOI:** 10.3389/fvets.2022.903890

**Published:** 2022-09-02

**Authors:** Atieh Darbandi, Shabnam Zeighamy Alamdary, Maryam Koupaei, Roya Ghanavati, Mohsen Heidary, Malihe Talebi

**Affiliations:** ^1^Department of Microbiology, School of Medicine, Iran University of Medical Sciences, Tehran, Iran; ^2^Department of Bacteriology, Faculty of Medical Sciences, Tarbiat Modares University, Tehran, Iran; ^3^Department of Microbiology and Immunology, School of Medicine, Kashan University of Medical Sciences, Kashan, Iran; ^4^Behbahan Faculty of Medical Sciences, Behbahan, Iran; ^5^Department of Laboratory Sciences, School of Paramedical Sciences, Sabzevar University of Medical Sciences, Sabzevar, Iran; ^6^Cellular and Molecular Research Center, Sabzevar University of Medical Sciences, Sabzevar, Iran

**Keywords:** brucellosis, vaccine, *Brucella*, vaccination, mouse

## Abstract

**Introduction:**

Despite the accessibility of several live attenuated vaccines for animals, currently, there is no licensed vaccine for brucellosis in human populations. Available and confirmed animal vaccines may be harmful and considered inappropriate for humans. Thus, human vaccines for brucellosis are required. We aimed to evaluate the effects of *Brucella* vaccines on mouse models and discuss the potential mechanisms of these vaccines for the design of the appropriate human vaccines.

**Materials and methods:**

A systematic search was carried out in Web of Science, Embase, and PubMed/Medline databases. The following MeSH terms were applied: brucellosis, vaccine, *Brucella*, and vaccination. The original manuscripts describing the *Brucella* vaccines on mouse models were included. The review articles, editorials, correspondences, case reports, case series, duplicate publications, and articles with insufficient data were excluded.

**Results:**

Of the 163 full texts that were screened, 17 articles reached to inclusion criteria. Combining the results of these trials revealed a reduction in bacterial load and colonization rate of *Brucella* in the spleen, an increase in inflammatory markers, especially IFN-γ and IL-4, and the highest levels of antibody classes in vaccinated animals compared to animals challenged with various virulent strains of *Brucella*. The majority of studies found that different anti-*Brucella* vaccines induced a significant protective effect in animals challenged with *Brucella* strains. Additionally, mice were given the highest level of *Brucella* vaccine protection and significant clearance of *Brucella* strains when the immunization was delivered *via* the IP (intraperitoneal) or IP-IN (intranasal) routes.

**Conclusion:**

Brucella is responsible for half-million new cases globally annually, and the lack of a proper human vaccine poses the risk of brucellosis. A variety of vaccines are used to prevent brucellosis. Subunit vaccines and recombinant human vaccines have higher safety and protective properties. Although vaccination helps brucellosis control, it does not eradicate the disease. Thus, we recommend the following strategies. (a) establishment of a registration system; (b) close monitoring of slaughterhouses, markets, and herds; (c) training veterinarians; (d) legal protection of the consequences of non-compliance with preventive measures.

## Introduction

Brucellosis is one the most prevalent zoonotic infectious diseases in the world, which affects abortion and infertility in domestic animals and is characterized with undulant fever and severe debilitating symptoms in humans. *Brucella* is considered as a class B bioterrorism due to its propensity for airborne transmission, its highly infectious nature, as well as its delayed diagnosis in favor of acquiring a chronic stage (1, 2). It also causes serious economic damage to the livestock industry due to offspring mortality, reduced milk production, and infertility. Evidence suggests that human brucellosis is associated with the disease persistence in livestock, emphasizing the importance of livestock vaccination as a controlling factor for brucellosis in animals and humans. Although S19, RB51, and REV-1, as currently licensed livestock vaccines, have been successfully used with 70% efficacy, they are inadequate for human use due to residual virulence that could result in the development of tmye disease (3). In the absence of a suitable human *Brucella* vaccine, animal vaccination is crucial not only to protect animal health but also to prevent zoonotic transmission. However, they have several disadvantages as follows: (i) they are still virulent for human, (ii) they are responsible for abortion when administered to pregnant animals, (iii) differentiate between infected and vaccinated animals is very difficult because they also induce a persistent serological response, and (iv) they are relatively unstable. Nevertheless, vaccination remains as the most successful and economic method for preventing and controlling brucellosis (3, 4). In order to avoid these drawbacks, alternative vaccination approaches or new generation of vaccines are needed. In developing countries, vaccination could be the only feasible strategy for brucellosis control programs. In fact, in areas with high prevalence or recurrence of brucellosis, vaccination of all animals over a short period of time is an effective management strategy. However, these vaccines have some drawbacks that make their use challenging; for example, S19 and Rev-1 could induce abortion in pregnant animals, and retention of their lipopolysaccharide (LPS) in vaccinated animals makes it difficult to differentiate vaccinated from naturally infected animals using serological methods (5, 6). These livestock vaccines are ~70% efficacious and are not considered safe to humans. At present, no vaccine is available for human use. Firstly, the residual virulence of live vaccines in humans and the abortifacient potential of smooth vaccines in pregnant animals make their use challenging. Also, the inability of these vaccines to induce an efficacious cross-protection against different *Brucella* species, affecting different animal species, limits their applicability (7, 8). Additionally, when S19 or RB51 vaccines are used in animals, the interference of vaccine-induced antibody response with conventional serological tests also creates problems during surveillance programs. Secondly, the production of these vaccines is complicated, and the development of an efficacious vaccine for brucellosis has been a challenge for scientists for many years. Therefore, the main focus of this review was to collect and compare different parameters affected by anti-*Brucella* vaccination in animal models and to investigate whether vaccination agents used in animal models could also be effective for humans.

## Materials and methods

The current study was carried out following the “Preferred Reporting Items for Systematic Reviews and Meta-Analyses” (PRISMA) statement (9).

### Search strategies

A comprehensive search was performed on the Web of Science, Embase, and PubMed/Medline databases to collect potentially relevant articles published from January 1, 2011 to March 25, 2021. The search was focused only on original articles published in English. The following keywords were used: “brucellosis,” “vaccine,” “vaccination,” “*Brucella*,” “*Brucella suis*,” “*Brucella melitensis*,” “*Brucella abortus*,” “*Brucella canis*”.

### Study selection

Research articles examining the *Brucella* vaccines on mouse models were included in this study. We excluded the original papers describing *in-vitro* or *ex-vivo* evaluations. Moreover, the review articles, editorials, correspondences, case reports, case series, duplicate publications, and articles with insufficient data were excluded. Selected studies were screened in two steps for eligibility. First, the title and abstract screening process was performed to identify articles possibly relevant to the research domain, then the full-text of those articles that seemed to meet the inclusion criteria was retrieved. Two authors independently checked the inclusion criteria in potentially relevant articles, and discrepancies between the authors were resolved by consensus discussion. Selected articles were then evaluated qualitatively.

### Quality assessment

Quality assessment was carried out on selected studies using the critical appraisal checklist provided by the Joanna Briggs Institute (JBI) (10). Eventually, eligible articles with high quality were selected and included in this research.

### Data extraction

The articles included in this research were subjected into data extraction process. The extracted data from the high-quality eligible articles were as follows: first author's name, published time, country, sample group, control measures, name of vaccine, type of vaccine, injection route, booster, adjutant and challenge outcomes.

## Results

**Figure 1:**
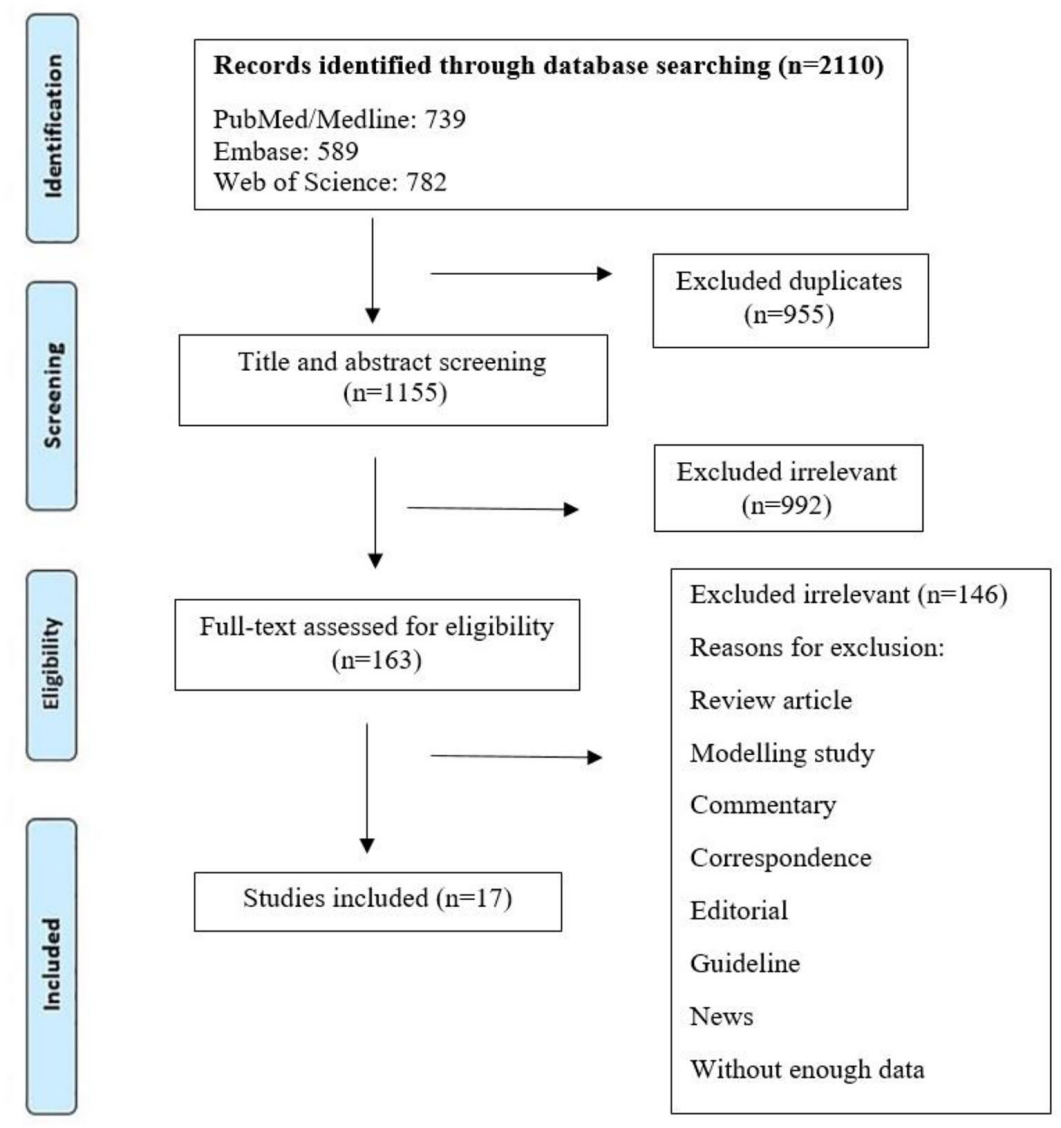
Flow diagram detailing the literature search and study selection.

Initially, 2110 references were found in the following databases: PubMed (*n* = 739), Web of Science (*n* = 782), and Embase (*n* = 589). A summary of the search strategy and study selection process is shown in Figure 1. By reviewing titles and abstracts, 1,118 articles were excluded for different reasons (duplicate studies as well as titles and abstracts irrelevant to the topic of this review), and 163 articles were retained for detailed full-text evaluation. Among which 146 articles were also excluded as irrelevant. Finally, 17 articles describing different attenuated and recombinant vaccines against brucellosis in animal models were selected and used in this research for further analysis. Among the 17 articles reviewed, two studies used live attenuated *B. melitensis* vaccines (4, 11), and two studies evaluated the effects of two new vaccine formulations against *B. melitensis* 16M, including live *Escherichia coli* expressing *Brucella* P39 protein combined with CpG oligodeoxynucleotides as well as recombinant invasive *E. coli* expressing *B. melitensis* outer membrane proteins (Omp31 or Omp16) and periplasmic protein BP26 (12, 13). Also, two studies investigated the effects of *Salmonella Typhimurium* (ST) vector vaccine and attenuated *Salmonella* strains secreting *Brucella* antigens (14, 15). The characteristics of the 17 studies included in this research are shown in Table 1.

**Table 1:** Characteristics of the included studies.

**References**	**Country**	**Publication Year**	**Sample group (wks. old)**	**Vaccine**	**Period (days)**	**Vaccine dose CFU/ml (volume μl)**	**Vaccine candidate**	**Challenge dose (CFU/mice; volume μl)**	**Control measures**	**Injection route**	**Booster**	**Adjutant**	**Outcome**
Alizadeh et al. (22)	Iran	2019	Female BALB/c mice (6–7)	*B. abortus*	44 days (28:imz/14:chg)	-	rOMP 16 kDa	*B. abortus* 544 or *B. melitensis* 16M (10^5^;-)	PBS	SC	Yes	Yes	rOmp16 is able to elicit efficient protective immune responses in animal hosts, increase IFN-γ and IL-4 levels, and increase total serum IgG level along with remarkable IgG1 and IgG2a responses.
Al-Mariri et al. (13)	Syria	2012	SPF female BALB/c mice (5)	Live *E. coli* expressing *Brucella* P39	Group 1: 68 (56:imz/12 chg) Group 2: 96 (84:imz/12 chg)	5 × 10^6^	*p39* gene of *B. melitensis*	*B. melitensis* 16M (5 × 10^4^;-)	PBS	IP	Yes	Yes (CpG oligonu cleotides)	This vaccine significantly reduced the number of *B. melitensis* 16M bacterial strains in mice spleens post-challenge infection, but this reduction was less than that induced by the Rev.1 vaccine.
Arenas-Gamboa et al. (25)	USA	2012	Female B6.129s2-*Irf1tm1 Mak*/J(6-8) mice	*B. melitensis* 16M *B. melitensis* 16M_vjbR *B. abortus* S19	67 (60:imz/7:chg)	1 × 10^6^	-	*B. melitensis* 16M biovar 1 (5 × 10^6^)	PBS	IP	-	-	Safety and protective efficacy of the vjbR mutant in an immunocompromised mouse model
				*B. abortus* S19_vjbR *B. abortus* 2308									
Curina et al. (11)	Italy	2018	Female BALB/c mice (4) and sheep (-)	Live attenuated REV1	90 day: mice 150 days: sheep	1 × 10^6^ (500) Mice: 1.6 × 10^9^/0.6 × 10^9^/0.8 × 10^9^/3.2 × 10^9^ Sheep: 1,000	-	-	Saline = mice Physiological solution = sheep	IP: mice SC: sheep	-	-	REV1 vaccine produced in bioreactor stimulated all immunocompetent cells tested, with a balanced innate and adaptive response in both sheep and mice.
Clapp et al. (24)	USA	2016	Female BALB/c mice: CD4^−/−^ and CD8^−/−^ C57BL/6 mice: IFN-γ^−/−^ BALB/c mice: (7–9)	BALB/c mice*:* ΔznuA *B. melitens* RB51, S19: IFN-γ^−/−^ mice: ΔznuA *B. melitensis*: CD4^−/−^ and CD8^−/−^ mice: ΔznuA *B. melitensis*, Rev-1	70 (42:imz/28:chg)	-	*-*	*B. melitensis* strain 16M *(-)*	PBS	NA	No	No	Stimulating systemic and mucosal immune protection *via* CD8+ T cell engagement
González-González et al. (21)	Mexico	2014	Female BALB/c mice (6–8)	RB51	77 weeks (56;imz/21:chg)	4 × 10^9^	Protoxin Cry1Ac of *B. thuringiensis*	*B. abortus 2308* (4 × 10^6^;-)	PBS	IN	Yes	Yes (Protoxin Cry1Ac)	The use of Cry1Ac protein as a mucosal adjuvant *via* the intranasal route could be a promising alternative to improve the current RB51 vaccine against brucellosis, with a more intense Th1-type response.
Gupta et al. (12)	India	2012	BALB/c female mice (4–6)	Invasive *E. coli* (DH5α)	63	2 × 10^7^ (200)	Omp31 or Omp16 or periplasmic protein BP26	*B. melitensis* 16M (2 × 10^4^;-)	Saline	IP	Yes		Invasive *E. coli* vaccines induce a cellular immune response and protect mice against infectious *B. melitensis* 16M challenge.
Jacob and Curtiss (17)	USA	2021	SPF female BALB/c (8)	Attenuated S19	56	7.3 × 10^6^ :IP (100) 8.5 × 10^6^:SC (100) 5.1 × 10^7^ :IN (25)	-	*-*	-	IP SC IN	-	-	S19 could be used safely and economically under BSL2 containment.
Jain et al. (26)	India	2015	Female Swiss albino mice (4–5	S19	45 days (30:imz/ 15:chg)	1 × 10^5^	Aluminum hydroxide gel Adjuvanted phage lysate of S19	*B. abortus* 544 (2 × 10^5^;-)	BSS	SC	-	Yes (PL-Plain or AD-Alum adjuvanted lysate)	Adjuvanted phage lysate exhibited the highest protective potency which was greater than that induced by standard S19 vaccine.
Jain-Gupta et al. (20)	USA	2012	Female BALB/c mice (6–8)	*B. melitensis* 16M	45 (30:imz/ 15:chg)	5 μg	OMP	*B. melitensis* (2 × 10^4^;-)	Saline	IP	Yes	Yes (Pluronic P85)	P85 enhanced the *in vitro* secretion of TNF-α by macrophages, enhanced the protection against *B. melitensis* challenge, and increased total IgG antibody production, but did not increase IFN-γ or IL-4 cytokine levels.
Lalsiamthara et al. (15)	Korea	2018	SPF female BALB/c mice (4)	Rough attenuated ST strain JOL1800 (JOL1878, JOL1879, JOL1880, and JOL1881) + LPS *Brucella*	45 (30:imz/ 15:chg)	2–8 × 10^7^ LPS: 5 μg	rB BLS Omp19 SOD PRAC SbA	*B*. *abortus* 544 (2 × 10^5^; 200)	PBS	IP IM SC OR	-	-	ST successfully induced an antigen-specific immune response. More effective protection was observed by inoculation *via* the IP and IM routes.
Minhas et al. (19)	India	2019	Male Swiss albino(8) mice	Recombinant *Brucella* Omp22	31	50 μg: recombinant proteins	rOmp22	-	rOmp22 alone	SC	Yes	FCA, FIA (control); rDnaJ9 (experiment)	rDnaJ from *Brucella* spp. could have immuno-modulatory properties and may stimulate both humoral as well as CMI arms of the immune system.
Mansour (23)	Saudi Arabia	2017	Female Bulb/C mice (7–8)	RB51	70	5 × 10^8^	-	*B. melitensis* bivar 3 (2 × 10^4^; 2,000)	RB51	IP	-	levamisole	Levamisole given 7 days or 0 and 7 days post-vaccination enhanced humoral and cell-mediated immune responses to RB51 strain (cytotoxic T cells).
Senevirathne et al. (14)	Korea	2019	SPF female BALB/c mic	Attenuated ST strain JOL1874 JOL1875 JOL1876 JOL1877	45 (30:imz/ 14:chg)	1 × 10^7^ (100)	SodC Omp19 BLS PrpA	*B. abortus* 544 (2 × 10^5^;-)	PBS	IP	-	-	Efficiently conferring a dual protection against both brucellosis and salmonellosis in immunized mice
Surendran et al. (16)	USA	2011	Female BALB/c mice (6–8)	Live attenuated RB51 Live attenuated Smooth S9 Rough RB51 RB51SOD RB51WboA Smooth *B. abortus* 2308	44 or 85 (42:imz/14 or 41:chg)	4 × 109 4 × 108	-	Smooth *B. abortus* strain 2308 (2 × 10^5^;-) (2 × 10^4^;-)	PBS	IN IP IM	Yes	-	IN administration of *B. abortus* vaccine strain 19 induced significant pulmonary clearance of *B. abortus* pathogenic strain 2308, but not rough RB51, RB51SOD, and RB51WboA vaccine strains. Neither strain RB51SOD nor strain RB51WboA induced significant improvement in pathogen clearance
													compared to strain RB51 upon IN vaccination.
Zhu et al. (18)	China	2016	Female BALB/c mice (6) Female guinea pigs (300–400 g)	Live attenuated S2	86 (56:imz/30 chg)	1 × 10^5^ (200)	-	*B. melitensis M* 28 *B. abortus*2308 *B. suis* S1330 (1 × 10^5^; 200)	Saline	SC	-	-	S2 vaccine stimulates good humoral and cellular immunity and protects animals against infection by heterologous, virulent *Brucella* species.
Zhu et al. (27)	USA	2011	Female BALB/c mice (4–5)	Live attenuated RB51 RB51SOD	42–49	4 × 10^8^ cells	-	-	Saline	IP	-	-	Higher cytotoxic T lymphocyte activity of RB51SOD rather than its parent strain RB51 due to the overexpression of Cu/Zn SOD and its release into extracellular area

Imz, immunization; chg, challenge; wks old, weeks old; SPF, specific pathogen free; rB, conserved Brucella antigens; BLS, Viz lumazine synthase; PRASbA, proline racemase subunit A; Omp19, outer membrane protein-19; SOD, Cu-Zn superoxide dismutase; IP, intraperitoneal; IM, intramuscular; SC, subcutaneous; IN, intranasal; OR, oral; NA, nasal; ST, Salmonella Typhimurium; PBS, phosphate buffered saline; BSS, buffered Brucella saline; rDnaJ, Brucella-specific recombinant HSP40.

Out of the 17 studies reviewed in this research, three studies (15–17) evaluated the effect of immunization route on protective efficacy of different *Brucella* vaccines. Jacob and Curtiss (17) revealed that the mean colonization titers of *B. abortus* S19, as a challenge strain for vaccine, in the spleen were higher in the IP (intraperitoneal) and SC (subcutaneous) groups during the first 14 days post-inoculation and then slowly decreased. On day 7, the mean CFUs (number of challenge strains) in the IP and SC groups were significantly higher than in the IN (intranasal) group (*p* < 0.001^**^). On day 14, the mean CFU was significantly higher in the IP group than in the SC group (*p* < 0.001^**^), as well as in the SC group than in the IN group (*p* < 0.001^**^). In the IN group, the mean CFU in the spleen was lower than in both lungs on day 7 but higher than in both lungs on day 21. Colonization titers in the spleen in the IN group were higher than in the IP and SC groups on day 28. This study showed that colonization titers in the spleen in the IP and SC groups peaked in the first 14 days of the experiment and then slowly decreased. In contrast, colonization titers in the IN group decreased during the first 14 days, but increased on day 21 and then slowly decreased. This may be due to that S19 bacteria probably needed more time to infect the spleen in the IN group. This study showed that S19 could infect and persist in mice tissues for at least 8 weeks after inoculation *via* the IP, SC, and IN administration routes. On the other hand, Lalsiamthara et al. (15) investigated the effect of immunization route on the protective efficacy of the combined LPS-*Brucella* vaccine. Their study results showed that the lowest CFU levels and the highest protection level were conferred to mice when the formulation was administered *via* the IP route. The difference between the SC/IP and IM (intramuscular) routes was not significant, but there was a significant difference between the SC and IP routes. Interestingly, immunization with the rough *Salmonella*-delivered recombinant combined LPS-*Brucella* vaccine *via* the IP or IM route produced significantly higher levels of protection compared to the commercial *B. abortus* strain RB51 vaccine *via* the IP route (*p* ≤ 0.005). Overall, mice groups immunized *via* the oral or IP route showed the production of specific IgG antibodies. Also, Surendran et al. (16) observed that single-dose IN vaccination with *B. abortus* strain RB51 and RB51SOD vaccines provided no protection against IN challenge with pathogenic strain 2,308 on day 14 or 41 in the lungs, spleen, or mediastinal lymph nodes (MLN). Besides, mice receiving IN booster dose showed no significant increase in bacterial clearance from the organs tested on both day 14 and day 41 compared to controls. There was no significant difference in clearance of strain 2,308 from the organs tested between various IN doses (10^7^, 10^8^, 10^9^ CFUs/mouse). Thus, IN vaccination had no protective efficacy. Also, none of the systemic vaccination routes induced significant clearance of strain 2,308 from the organs tested compared to PBS (phosphate buffered saline) control. Furthermore, regarding the clearance of strain 2,308 from the spleen, lungs, or MLN, there was no significant difference between different vaccination routes. Besides, no significant improvement in bacterial clearance from the lungs or MLN was obtained with IP-IN combination vaccination strategy. Only IP-IN administration of the vaccine strain RB51SOD induced significant clearance of strain 2,308 from the spleen (*p* = 0.0146). Finally, IM and ID (intradermal) systemic priming followed by IN booster provided no protection in mice against IN pathogenic strain 2,308 infection in the organs tested.

Eight out of the 17 studies (12, 14, 15, 18–22) investigated antibody classes' responses in experimental groups (a summary is provided in Table 2). In all the eight studies reviewed, the maximum levels of antibody classes were observed in vaccinated groups. Mansour (23) showed that levamisole treatment either 7 days or 0 and 7 days post-vaccination by RB51 elicited a strong immune potentiating effect, so that the mean antibody levels measured by ELISA throughout the experimental period were higher in levamisole-treated mice compared to levamisole-untreated vaccinated mice. Mice treated with levamisole simultaneously (day 0) with vaccination showed a mild elevation in antibody titer compared with vaccinated mice receiving no treatment. The highest level of delayed-type hypersensitivity (DTH) was observed in the group of mice treated with levamisole 0 and 7 days post-vaccination, followed by the group of mice treated with levamisole 7 days post-vaccination and then the group of mice treated with levamisole simultaneously with vaccination.

**Table 2:** The antibody responses in experimental studies.

**References**	**Vaccine**	**IgG total**	**IgG1**	**IgG2**	**IgM**	**sIgA**
Zhu et al. (18)	Live attenuated *B. suis* strain 2 (S2)	↑	-	-	-	-
González-González et al. (21)	Protoxin Cry1Ac of *Bacillus thuringiensis* with RB51 vaccine strain of *B. abortus*	↑	↓	↑	↑	-
Alizadeh et al. (22)	Outer membrane protein of *B. abortus* (Omp16)	↑	↑	↑	-	-
Jain-Gupta et al. (20)	Pluronic P85 as an adjuvant to enhance the efficacy of *B. melitensis* OMVs	↑	-	-	-	-
Gupta et al. (12)	Recombinant inv- *E. coli* expressing Omp31 or 16 and BP26 *B. melitensis* proteins	-	↑	-	-	-
Minhas et al. (19)	Recombinant HSP40 (rDnaJ) co-immunization with *Brucella* rOmp22	-	↑	↑	-	-
Lalsiamthara et al. (15)	*Brucella* antigens delivered by *S. Typhimurium* (ST) vector	↑	-	-	-	-
Senevirathne et al. (14)	Attenuated *Salmonella* strains secreting *Brucella* antigens SodC, Omp19, BLS, and PrpA	↑	-	-	-	↑

Based on Table 3, among the reviewed articles, 10 studies (12–15, 18–22, 24) investigated some inflammatory markers (IFN-γ, IL-4, IL-5, IL-12, IL-17, IL-22, IL-23, TNF-α) in animals immunized with anti-*Brucella* vaccines and analyzed cytokine responses to immunization.

**Table 3:** The cytokine responses in experimental studies.

**References**	**IFN-γ**	**IL-4**	**IL-5**	**IL-12**	**IL-17**	**IL-22**	**IL-23**	**TNF-α**
Al-Mariri et al. (13)	↑	-	-	-	-	-	-	-
Lalsiamthara et al. (15)	-	-	-	-	↑		↑	-
Zhu et al. (18)	↑	-	-	-	-	-	-	↑
González-González et al. (21)	↑	-	-	-	-	-	-	↑
Alizadeh et al. (22)	↑	↑	-	-	-	-	-	-
Gupta et al. (12)	-	-	-	-	-	-	-	-
Clapp et al. (24)	↑	-	-	-	↑	↑	-	-
Jain-Gupta et al. (20)	↑	-	-	-	-	-	-	-
Minhas et al. (19)	-	↑	-	↑	-	-	-	-
Senevirathne et al. (14)	↑	↑	-	-	-	-	-	-

Among the 17 studies reviewed, 13 articles (12, 14–18, 20–22, 24–27) reported the induction of a significant protective effect in vaccinated animals challenged with different virulent strains of *Brucella*. In these studies, different anti-*Brucella* vaccines conferred significantly higher levels of protection in vaccinated mice compared to saline-vaccinated mice or control groups. Also, two studies (21, 26) showed a decrease in bacterial load and colonization in the spleen. González-González et al. (21) showed that co-administration of pCry1Ac (adjuvant) with RB51 conferred a significant level of protection against IN challenge with virulent strain *B. abortus* 2,308, thereby reducing the spleen colonization level by 1.0 log_10_, which was significantly different compared to both groups of PBS control (*p* < 0.05) and RB51 alone (*p* < 0.05). Besides, Jain et al. (26) reported that on day 15 post-challenge, all lysate (plain and alum-adsorbed)-vaccinated groups showed a significant (*p* < 0.05) reduction in bacterial load or total viable counts (TVC) in the spleen [protective values (Y) from 3.47 to 1.80] compared to the unvaccinated control group (*Y* = 4.81), confirming the dose-dependent protective efficacy of phage lysate. Also, one week post-challenge (9 weeks post-vaccination), a marked decrease in splenic and hepatic bacterial loads (4-log-unit reduction) was observed in mice vaccinated with the 16MΔvjbR mutant or S19ΔvjbR mutant.

## Discussion

*Brucella* is annually responsible for 500,000 new brucellosis cases worldwide (28). Due to its pathogenic nature for humans, communicability, moderate side effects, and potential for use as a bioterrorism agent, *Brucella* has been introduced by the CDC as a category B agent that should be used under level 3 biosafety (17). The lack of a proper human vaccine poses the risk of developing brucellosis as a bioterrorism weapon (28). The purpose of this systematic review was to evaluate different vaccines designed against brucellosis in mouse models and their effectiveness in order to paw the way for the design and manufacture of appropriate human vaccines.

So far, various brucellosis eradication methods have been proposed, including live vaccines such as S19 and Rev1, extensive protective coverage, appropriate diagnostic tests, continuous removal of infected livestock, and restriction of animal movement from infected to free herds (29). Due to the economic losses associated with common infections between humans and animals (30), research teams are looking for new vaccines, such as subunits vaccines (31), bacterial vector-based vaccines (32), and vaccines based on overexpression of protective homologous antigens (33). One of the reasons for not using new vaccines is the resistance of government regulators in some countries such as developing countries (34).

So far, a variety of vaccines have been used to prevent brucellosis. Common types of vaccines include smooth vaccines and rough vaccines. Other types of vaccines include vector-delivered *Brucella* vaccines, genetically engineered attenuated vaccines, and subunit vaccines. Subunit vaccines are divided into two categories, including recombinant protein- and DNA-based vaccines (34). Vaccination has several benefits, such as limiting *Brucella* infection, limiting shedding, disrupting animal-to-animal transmission, and reducing the spread of disease between animals and humans (29). If vaccination is accompanied by the removal of infected animals, it could lead to the selection of more resistant strains of the disease (35). Dysfunctional vaccines have some disadvantages. In fact, vaccinated animals act as a source of infection and transmit malignant *Brucella* strains (36). The use of low-performance *Brucella* vaccines creates a false sense of security among ranchers and health officials, and may lead them to believe that the herd is fully protected (36).

Subunit vaccines and recombinant human vaccines have high safety and protective properties. The formulation of subunit vaccines should be optimized (for example, a suitable adjuvant should be used). In live recombinant human vaccines, the use of a vaccine vector is important (37). Vaccination of livestock is the most important way to prevent brucellosis until appropriate human vaccines are discovered (37). So far, different vaccines have been designed for human use, but each has disadvantages that make its use challenging. For example, strain 19-BA, which is applied intradermally by scarification, provides limited protection for a relatively short period of time and requires re-immunization. Another disadvantage is the occurrence of hypersensitivity reactions. *Brucella abortus* 84-C and M-104 are used intradermally or as aerosols, which seem to be effective but could cause severe reactions if not used properly or given to sensitive people (38).

*Brucella* DNA-based vaccines are a type of subunit vaccines that could induce humoral and cellular immune responses after repeated use (39). DNA vaccines are plasmids that express the gene encoding a specific antigen. Among the most common genes used are L7/L12, BLS, BCSP31, SOD Cu/Zn, Omp16, P39, and BAB1-0278. Adjuvant is not commonly used in DNA vaccines (3). The DNA vaccine encoding BAB1-0278 protects mice against *B. abortus* (40). DNA vaccines containing BAB1 0273 and/or BAB1 0278 and SOD C elicit immune responses in mice, but have low protective effect (41). DNA vaccines encoding p39 and/or groEL as well as other DNA-based vaccine candidates require several booster vaccinations while providing low levels of protection. Therefore, more studies are needed in this area (39). Two methods could be used to enhance immune responses and the efficacy of *Brucella* DNA vaccines, including the expression of several antigens in DNA vaccines (Omp16 and L7/L12), and the expression of cytokines as adjuvants (SOD with IL-18 or IL-12) (3). DNA vaccines has been studied more in mouse models than in other natural hosts (42). DNA vaccines must be injected intramuscularly, which requires large amounts of DNA. Gene guns and nanoparticles could be used to solve this problem. These methods require less DNA and increase cell uptake and DNA half-life (34). The advantages of using these vaccines include long-term expression, better stability, safe vaccination, and easy production (34). DNA vaccines are suitable for those diseases against which cellular immunity is required to defend the host. These vaccines have been used in several human studies and shown to induce weaker immune responses in humans than in mice; thus, they need to be optimized. But optimization increases pro-inflammatory reactions (42). Among the new optimization methods that could be used for this purpose are two innovative methods of delivery and codon optimization (34).

In some vaccines, CpG oligonucleotides (CpG ODN) containing unmethylated CG motifs similar to those found in bacterial DNA stimulate toll-like receptors (TLRs) in vertebrates, especially TLR9 (43). Stimulation of these receptors triggers innate, humoral, and cellular immune responses (44). CpG ODN has various effects on the immune system, such as accelerating and enhancing antigen-specific immune responses, inducing Th1 and pro-inflammatory cytokines, as well as supporting the maturation and inactivation of professional antigen presenting cells (APCs). CpG ODN is safe when administered to humans as an adjuvant and may support enhanced vaccine-specific immune responses (45).

Dual vaccines, which are used against two pathogens, have also been tested against brucellosis. So far, dual vaccines against *Brucella* and two pathogens of *Helicobacter pylori* (46) and *E. coli* O157:H7 (47) have been used in mice. In all of these vaccines, good efficacy and immune responses have been observed. Another uncommon vaccine is the ghost vaccine, which uses *Brucella* lysed extract to create immunity. The ghost vaccine has significant protective effects on the mouse host. Due to the beneficial effects of such vaccines, it is recommended that further studies be performed on them.

It is difficult to obtain a suitable vaccine that could elicit protective responses against inhaled *Brucella* infection (48). Some of the reasons for the difficulty in making this kind of vaccine could be as follows. First, tested vaccines are unable to elicit an innate and efficient immune response in the lung microenvironment. Second, live *B. abortus* vaccines suppress innate immune responses and affect DCs ability to induce protective cellular immunity. Third, the intracellular nature of *Brucella* makes the vaccine unable to induce efficient acquired immunity. After inhalation, *B. abortus* multiplies in lung epithelial cells and alveolar macrophages without eliciting a strong innate immunity. Therefore, it causes the bacteria to escape from the identification and purification mechanisms of acquired immunity (48). It has been shown that subcutaneous (SC) administration of Rev1 reduces the bacterial load in the spleen of *B. melitensis* 16M-infected mice, but has no effect on the bacterial load in the liver and lungs. IN administration of the Rev1 vaccine reduces *B. melitensis* 16M load in the lungs and spleen (49).

In some of the studies reviewed in this research, weight gain or loss in organs such as the spleen was reported. Weight gain may be due to increased inflammation because the immune system responds to the presence of *Brucella* (17).

*Brucella* infection occurs in host cells. Thus, infected cells must kill either the bacteria or themselves. In this case, antibody-mediated immunity could be used to clear the bacteria (50). Once *Brucella* enters the host cell, the bacteria are killed, and the bacterial peptides are processed on the surface of the APCs. These peptides are associated with MHC I and MHC II. Peptide-MHC is detected *via* TCR (T-cell receptor). CD${}_{4}^{+}$ T cells recognize peptide-MHC II, and CD${}_{8}^{+}$ T cells recognize peptide-MHC I (50).

Immunity to *B. abortus* is induced in a variety of ways, including activation of antigen-specific T cells, humoral responses, CD${}_{4}^{+}$ T cells, and CD${}_{8}^{+}$ T cells (50). To activate innate immunity, pathogen-associated molecular patterns (PAMPs) are first identified by TLRs, which then activate APCs. As a result, bacterial phagocytosis is facilitated (50). Specific subtypes of IgG, such as IgG2a and IgG3, are produced in humans in humoral responses to intracellular brucellosis (50). An increase in immunoglobulin levels means that the vaccine has a protective effect. In the present study, the highest increase in antibody was related to IgG total. Primary effective immunity in mice is mediated through the production of IL-12-dependent IFN-γ by CD${}_{4}^{+}$ T and CD${}_{8}^{+}$ T cells, which results in nitric oxide-dependent killing of bacteria by infected macrophages (51). It has been observed that in Balb/c mice, *Brucella* susceptibility is caused by an increase in the levels of IL-4 and IL-5 produced by Th2 (50). Among the reviewed articles in this study, IFN-γ levels were measured in most studies, which also showed the highest elevation. Other cytokines (e.g., IL-4, IL-5, and IL-17) were less studied or not studied at all.

The ability of *B. abortus* to produce antigens independent of T-helper cells as well as CTL (cytotoxic T lymphocyte) responses independent of CD${}_{4}^{+}$ T cells could be used to make vaccines (50). By conjugating peptides and proteins to *B. abortus*, antibody and CTL responses are evoked in the absence of CD${}_{4}^{+}$ T cells. This approach has been used successfully in mice and monkeys to produce systemic and neutralizing mucosal antibodies (50).

Vaccination helps control brucellosis but does not eradicate it. With 80% vaccination coverage, good results could be achieved (52). Some additional measures to eradicate *Brucella* are as follows: (1) permanent animal identification and registration system for close monitoring; (2) close monitoring of slaughterhouses, markets, and herds for timely identification of infected animals; (3) continuous monitoring of herd movements to prevent infection and its spread; (4) training veterinarians and supervisors; (5) compensation for farmers' financial losses for the removal of contaminated animals; and (6) legal protection of the consequences of non-compliance with formal preventive measures (53).

The future of developing a new brucellosis vaccine depends to some extent on new scientific developments in the field of diagnostic tools. Considering the recent advances in the field of post-genomics, the completion of the human and mouse genome projects provides a golden opportunity to screen genomic responses to *Brucella* infections in the host. In addition, the use of diagnostic systems based on fluorescence or luminescence with the help of a computer can be used to monitor the position of *Brucella in vitro* and *in vivo*. New *Brucella* vaccines should be developed based on a proper understanding of bacterial and host pathogenesis. *Brucella* vaccine research can be accelerated by using signature-tagged transposon mutagenesis (STM), green fluorescent protein (GFP)-expressing *Brucella* strains, and knockout (KO) mice. By using the technique of extensive genome screening, *in vitro* and *in vivo* imaging of bacteria and KO mice, the detection of weakened strains is facilitated and the speed of vaccine production is accelerated (54). In order to produce *Brucella* vaccine, it is necessary to pay attention to the following points: first, to go through the necessary steps to issue a license, second, to evaluate the effectiveness of the vaccine in two animal models (one a small animal, such as a mouse, and the other a larger animal, such as a non-human mammal), and third, determine the safety, immunogenicity, and efficacy of the vaccine. If in some cases it is not possible to check and show the protective efficiency clearly in humans, then the effectiveness should be predicted (8).

The limitations of this study are as follows. First of all, the information available in the studies in this systematic review was incomplete in some areas, for example, all the studies that were included in this report did not determine the type of cytokine or the type of immunoglobulin, and this caused the lack of a comprehensive and accurate analysis. Second, different types of vaccines were used in various studies to protect and prevent *Brucella* infection, which led to the dispersal of the contents. It is recommended that other researchers focus on each vaccine separately to obtain a detailed analysis. Third, in this study, only studies that were published in English were included, which may have caused some data to be lost.

Since *B. melitensis* is the most common *Brucella* species, a live human vaccine based on this bacterium should be developed (55). Finally, to answer the question of which type of vaccine is the best option for the production of Brucellosis vaccine, one should pay attention to the specific characteristics of each type of vaccine. Although the live attenuated vaccine has good immunogenicity and protective effect, it is difficult to obtain a license for its use. Recombinant vaccines perform very well in human clinical trials and can be a suitable alternative. A subunit vaccine induces immunity, but multiple subunits may be required to produce high protection. Vaccines based on live *Brucella* are expensive because they have to be contaminated at a high level. Vaccines based on purified proteins are not suitable for brucellosis endemic areas because they require a specialist and a refrigerator. Therefore, in such a situation, vector-based vaccines such as DNA vaccines or live attenuated vaccines can be the best option because they enable the transfer of several protective subunits, can be used without injection, are fast and affordable (8).

## Author contributions

AD, SA, MK, RG, MH, and MT contributed in revising and final approval of the version to be published. All authors agreed and confirmed the manuscript for publication.

## Conflict of interest

The authors declare that the research was conducted in the absence of any commercial or financial relationships that could be construed as a potential conflict of interest.

## Publisher's note

All claims expressed in this article are solely those of the authors and do not necessarily represent those of their affiliated organizations, or those of the publisher, the editors and the reviewers. Any product that may be evaluated in this article, or claim that may be made by its manufacturer, is not guaranteed or endorsed by the publisher.
